# What Is Your Diagnosis? Splenic Mass in an African Pygmy Hedgehog

**DOI:** 10.1111/vcp.70021

**Published:** 2025-08-21

**Authors:** Gabriela Hartmann, Milena Carolina Paz, Paola Sônego, Ana Carolina Contri Natal, Lívia Surita, Marcele Bettim Bandinelli, Luciana Sonne

**Affiliations:** ^1^ Department of Veterinary Pathology, Faculty of Veterinary Medicine Federal University of Rio Grande do Sul Porto Alegre Rio Grande do Sul Brazil; ^2^ Department of Animal Medicine, Faculty of Veterinary Medicine, Hospital of Veterinary Clinics Federal University of Rio Grande do Sul Porto Alegre Rio Grande do Sul Brazil

## 1 Case Presentation

A 5‐year‐old male African pygmy hedgehog ( *Atelerix albiventris* ) was presented to a veterinary teaching hospital with a 7‐day history of prostration. After 2 days of anorexia, the patient died, and the corpse was sent to the Department of Veterinary Pathology of the Federal University of Rio Grande do Sul. Postmortem examination revealed marked diffuse splenomegaly. The spleen had rounded edges, measured 7 × 2.3 × 1.5 cm, and there were multiple white, elevated, soft, often coalescent masses (Figure 1). Fine‐needle aspirate samples were collected from the spleen in less than 1 h after the hedgehog's death, and the smears were stained with an aqueous Romanowsky stain (Panótico Rápido) for the cytologic evaluation (Figure 2). Additional gross findings included a 0.5 cm diameter white firm nodule in the right medial hepatic lobe and multiple white, firm, up to 1 mm nodules in the lungs. Tissue samples of the spleen, liver, gallbladder, kidneys, stomach, small and large intestines, adrenal glands, urinary bladder, pancreas, bone marrow, lungs, heart, trachea, esophagus, thyroid gland, and encephalon were collected for histopathology.

**FIGURE 1:**
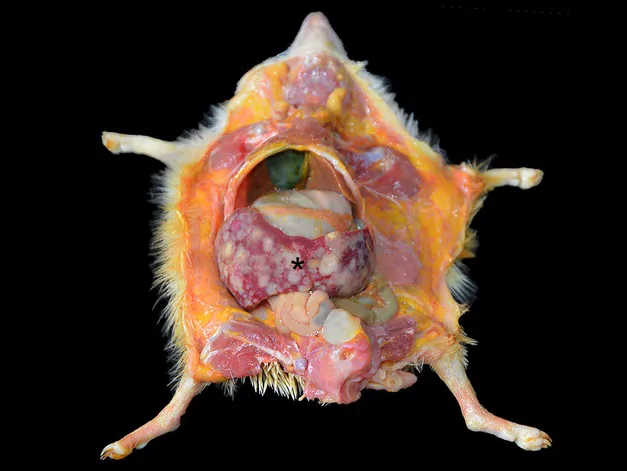
A gross image of the spleen, submitted for cytologic and histopathologic evaluation, of an African pygmy hedgehog. The spleen is markedly enlarged with multifocal to coalescent white, soft, elevated masses (asterisk).

**FIGURE 2:**
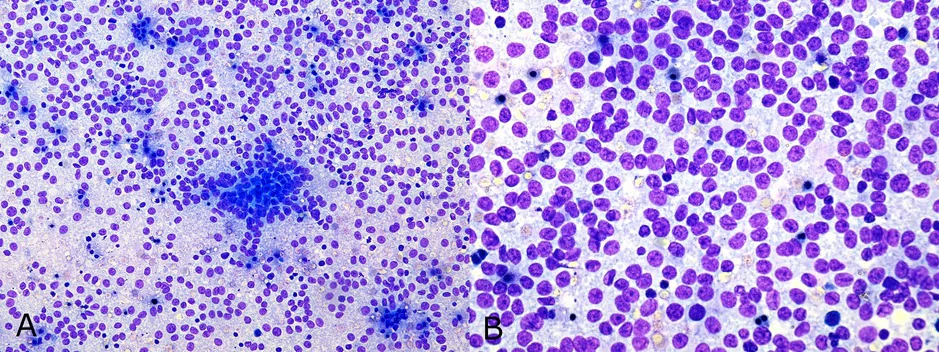
Photomicrographs of an impression smear from a splenic mass in an African pygmy hedgehog. Aqueous Romanowsky stain. (A) ×20 objective. (B) ×40 objective.

### 1.1 Cytologic Interpretation

Spleen, neuroendocrine neoplasia.

### 1.2 Cytologic Description

Cytologic preparations were highly cellular and composed predominantly of multiple naked nuclei mixed with fewer round to polygonal intact cells exfoliating individually and less frequently in disorganized, loosely cohesive clusters and acinar‐like structures. When assessable, cellular borders were mildly discrete, and the cells had a high nuclear‐to‐cytoplasmic ratio and a small amount of lightly basophilic and finely granular cytoplasm. The nuclei were round to oval, frequently indented, with a finely stippled chromatin pattern and 1–2 prominent nucleoli. Anisocytosis was not assessable; anisokaryosis was marked, and there were occasional binucleated cells and nuclear molding. Occasionally, there was a linear arrangement of neoplastic nuclei (palisading). A few apoptotic cells were interspersed with the neoplastic cells. There were 5 mitotic figures observed in 2.37 mm^2^ (equivalent to 10 FN22/40× fields). Admixed with the neoplastic cells, there were few plasma cells, lymphocytes, erythrocytes, and scant neutrophils, interpreted as normal splenic parenchyma, and erythroid precursors, interpreted as extramedullary hematopoiesis, in a finely granular basophilic background.

## 2 Additional Results

On histopathology, the splenic parenchyma was almost completely effaced by a poorly demarcated, unencapsulated, neoplastic proliferation of neuroendocrine‐type cells arranged in packets, separated by a delicate fibrovascular stroma (Figure 3). Neoplastic cells were polygonal, with a moderate amount of finely granular, lightly eosinophilic cytoplasm with indistinct cell borders. The nuclei were round to oval, with finely stippled chromatin and 1–2 prominent nucleoli. Anisocytosis was mild, anisokaryosis was moderate, and 12 mitotic figures were seen in 2.37 mm^2^. In the adjacent parenchyma, there were scattered areas of necrosis, foci of extramedullary hematopoiesis, and hemorrhage. In the liver and lung sections, there were multifocal areas of similar neoplastic proliferation (Figure 3).

**FIGURE 3:**
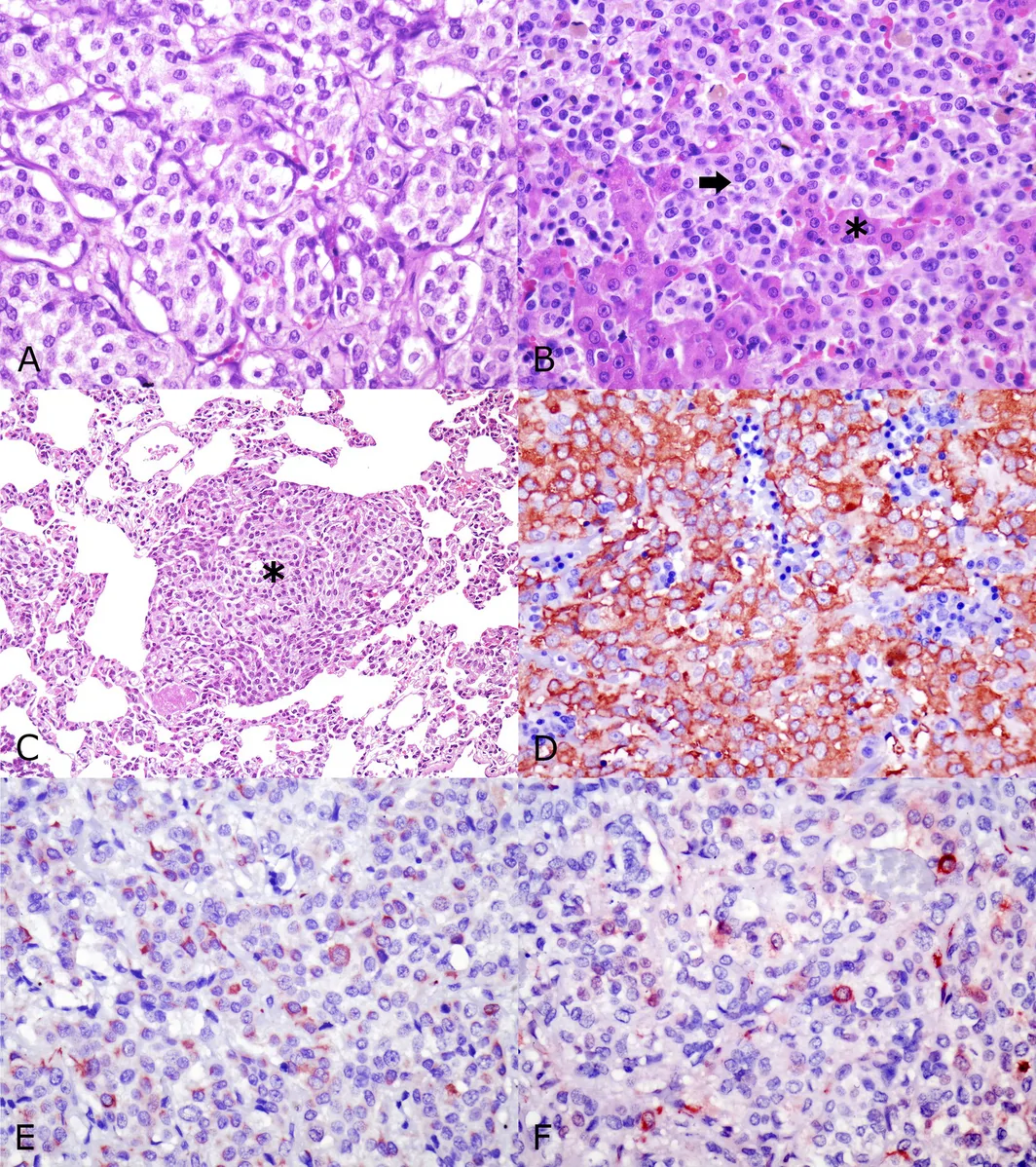
Photomicrographs of histologic sections from a splenic mass, liver, and lung metastasis in an African pygmy hedgehog. (A) The spleen is almost completely effaced by a neoplastic proliferation composed of neuroendocrine cells arranged in packets, sustained by delicate fibrovascular septa. H&E; ×40 objective. (B) In the liver sections, hepatocyte cords (asterisk) were frequently interrupted by clusters of neoplastic neuroendocrine cells (arrow). H&E; ×40 objective. (C) In the lungs, there were multifocal microscopic nodules (asterisk) composed of similar neoplastic neuroendocrine cells. H&E; ×20 objective. (D) Right panel indicates intense and diffuse cytoplasmic immunoreactivity within neoplastic splenic cells; 40× objective; mouse anti‐synaptophysin/AEC. (E) Left panel indicates moderate and multifocal cytoplasmic immunoreactivity within neoplastic splenic cells; 40× objective; mouse anti‐pan‐cytokeratin/AEC. (F) Right panel indicates weak‐to‐moderate and multifocal cytoplasmic immunoreactivity within neoplastic splenic cells; ×40 objective; mouse anti‐neuron‐specific enolase/AEC.

Multiple spleen, liver, and lung sections were submitted to immunohistochemistry using a monoclonal mouse anti‐human pan‐cytokeratin antibody (Dako; AE1/AE3; ready‐to‐use), a monoclonal mouse anti‐human vimentin antibody (Invitrogen; V9; 1:200), a monoclonal mouse anti‐human synaptophysin antibody (Dako Omnis; DAK‐SYNAP; ready‐to‐use), and a monoclonal mouse anti‐human neuron‐specific enolase antibody (DakoCytomation; BBS/NC/VI‐H14; 1:200). The antibodies were validated in the species with internal positive controls. Normal hedgehog's pancreas and adrenal gland sections were used for the neuroendocrine markers and lung sections for pan‐cytokeratin and vimentin. About 40% of the neoplastic cells from the splenic mass and lung and liver metastases expressed moderate, focal cytoplasmic pan‐cytokeratin immunolabeling (Figure 3), over 95% of the cells showed intense cytoplasmic synaptophysin immunolabeling (Figure 3), and about 30% presented weak‐to‐moderate cytoplasmic neuron‐specific enolase immunolabeling (Figure 3). The definitive diagnosis was a splenic metastatic neuroendocrine malignant tumor (carcinoid).

## 3 Discussion

Neoplastic diseases are among the most commonly reported diseases in African pygmy hedgehogs [1, 2, 3]. Retrospective necropsy studies suggest that up to 53% of the causes of death in the species are related to neoplasia [1], and 85% of those neoplasms are malignant [2, 3]. Aging is the main risk factor associated with neoplasia in hedgehogs, and the median age for the diagnosis is 3.5 years [1]. The most often affected organs belong to the integumentary, hemolymphatic, digestive, endocrine, and reproductive systems, with cytology being a useful diagnostic tool for these neoplasms [1, 3].

Malignant neuroendocrine neoplasms (carcinoids) arise from disseminated neuroendocrine cells in various organs, like the gastrointestinal tract, nasal cavity, liver, gallbladder, and skin [4, 5]. In hedgehogs, carcinoids can also arise from the spleen, although the organ does not normally contain disseminated neuroendocrine cells [5]. It is hypothesized that those tumors may originate from aberrant or ectopic neuroendocrine tissue in the spleen of the species [5]. Cytologic preparations of neuroendocrine tumors are typically associated with exfoliation of artifactual naked nuclei because of their fragile cytoplasm. Admixed with those nuclei, a few round to polygonal cells with intact moderate to abundant lightly basophilic cytoplasm and intracytoplasmic basophilic granules may be observed [4].

In our case, a diagnosis of neuroendocrine malignant neoplasia was made based on cytologic, histopathological, and immunohistochemical findings. Additionally, to reinforce the diagnosis, there were no gross or histologic alterations in organs in which neuroendocrine tumors typically arise (e.g., pancreas, thyroid, adrenal glands, intestines, gallbladder). Grossly and cytologically, neuroendocrine tumors may resemble lymphomas [4]. That differentiation is essential in the spleen, where, in hedgehogs, lymphomas, and myeloid leukemia are much more frequently associated with splenomegaly than neuroendocrine tumors [2, 3]. Cytologically, lymphomas are predominantly composed of a uniform and dense population of round cells that may also exhibit naked nuclei in smear slides due to cellular fragility. However, in lymphomas, nuclear streaming formation as a distention/pressure artifact helps in this differentiation [6]. Additionally, lymphomas have scant to moderate amounts of dark blue cytoplasm, with one to multiple nucleoli, high mitotic activity, and lymphoglandular bodies [3, 4]. Hence, cytology helps differentiate these conditions.

Histopathology, as well as in other neuroendocrine neoplasms, reveals packets, nests, rosettes, or pseudorosettes of neuroendocrine cells separated by delicate fibrovascular septa; the neoplastic cells have finely granulated cytoplasm [5]. Synaptophysin, neuron‐specific enolase, and chromogranin A expression in immunohistochemistry confirm the neuroendocrine origin [4, 5]. In our case, chromogranin A was not performed, but synaptophysin and neuron‐specific enolase immunostaining confirmed the neuroendocrine origin; pan‐cytokeratin immunostaining was useful to confirm the epithelial origin. Overall, our case demonstrates that neuroendocrine tumors should be considered a differential diagnosis in splenic masses when there is exfoliation of naked nuclei in cytologic samples and highlights the cytologic features of an underexplored neoplastic process of the spleen in a hedgehog.

## Conflicts of Interest

The authors declare no conflicts of interest.
